# No new evidence for an Atlantic eels spawning area outside the Sargasso Sea

**DOI:** 10.1038/s41598-022-14882-8

**Published:** 2022-07-12

**Authors:** Reinhold Hanel, Lasse Marohn, Håkan Westerberg

**Affiliations:** 1 Thünen Institute of Fisheries Ecology, Herwigstraße 31, 27572 Bremerhaven, Germany; 2 Swedish University of Agricultural Sciences, Department of Aquatic Resources, Institute of Freshwater Research, 178 93 Drottningholm, Sweden

**Keywords:** Animal migration, Marine biology, Behavioural ecology

**arising from**: Y.-L. K. Chang et al.; *Scientific Reports* 10.1038/s41598-020-72916-5 (2020).

## Introduction

The Sargasso Sea was identified as the spawning area of the European eel (*Anguilla anguilla*) 100 years ago, and numerous subsequent surveys have verified that eel larvae just a week old are regularly recorded there. However, no adult eels or eel eggs have ever been found, leaving room for alternative hypotheses on the reproduction biology of this enigmatic species. Chang et al.^1^ theorize about an area along the Mid-Atlantic Ridge as a potential spawning ground. The main argument for this hypothesis was that the chemical signature found in eel otoliths would indicate that early stage larvae had been exposed to a volcanic environment, such as the one present along the Mid-Atlantic Ridge. Since this correlation was solely based on a mis-interpretation of cited literature data, no new, conclusive information to pinpoint the Mid-Atlantic Ridge as an additional or even alternative spawning area was presented by Chang et al.

For more than 100 years, the life history of Atlantic eels remains a matter of scientific debate. In a recent paper by Chang and colleagues, published in *Scientific Reports* (Sci Rep 10, 15981 (2020)), it is hypothesized that the spawning areas of the European eel (*Anguilla anguilla*) and the American eel (*A. rostrata*) are located along the Mid-Atlantic Ridge at longitudes between 50° W and 40° W^1^. This area lies outside the Sargasso Sea, which has so far been widely assumed to be the spawning region of both species since the beginning of the twentieth century^2^. The Danish researcher Johannes Schmidt collected eel leptocephali 30 mm long or less, some as short as 9 mm, all south of 30° N and west of 50° W^3,4^. Since then, Schmidt’s assumption was supported by a number of investigations that found recently hatched European eel larvae (< 12 mm) in a 2000 km wide region from 70° W eastward to 50° W (e.g.^5–8^).

Nonetheless, final evidence about the precise locations of specific spawning sites and the timing of spawning is still lacking and information on hydrographic conditions at the depth and place of spawning also remains limited^9^.

In their paper, Chang et al. raised four arguments to support a Mid-Atlantic Ridge hypothesis: (1) The importance of seamounts as orientation and navigation cues towards a spawning area, (2) The results of a drift simulation showing that the distribution of virtual eel larvae that were hatched at the Mid-Atlantic Ridge would result in the observed distribution of eel larvae in the Atlantic Ocean, and (3) elevated manganese (Mn) concentrations in the otolith cores of glass eels caught in western European estuaries compared to those of leptocephalus larvae caught in the Sargasso Sea, which the authors interpret as a hint for successful spawning only in areas with volcanic activity. In addition, the authors (4) mention insufficient sampling efforts and a limited area coverage of recent surveys as a possible reason for “false negative” observations along the Mid-Atlantic Ridge.

However, none of the arguments seem to withstand careful scrutiny.

## The importance of seamounts as orientation and navigation cues towards a spawning area

The idea that eels would need sea mounts or other underwater elevations as landmarks, signposts or even meeting places to enable orientation during their migration over thousands of kilometres in an otherwise supposedly amorphous mass of water has a long history. Different authors tried to verify a seamount hypothesis in the Atlantic Ocean. Among these, even the so-called *Echo Bank*, a fictive sea mount, reported more than 100 km southeast of the main concentration of larvae (see^10^) to elevate up to 60 m below the surface in 1937 and 1946 (see^11^), was long discussed as a possible spawning site^12^ or solid reference point for orientation^13^. However, the existence of this seamount could not be confirmed in later expeditions^11^.

Chang et al.^1^ now argue that the Mid-Atlantic Ridge in its north–south orientation may form a Y-axis, which together with hydrographic fronts as X-axis could act as a signpost for the eels. A problem with this hypothesis is that no clear mechanism is proposed for how the migrating eels can detect the ridge. According to recent bathymetric data with 15 arc-second resolution (GEBCO 2020 Grid https://doi.org/10.5285/a29c5465-b138-234d-e053-6c86abc040b9) the highest peaks are well below the maximum migration depth of the eel, which is 800–1000 m^14,15^. Magnetic anomalies are suggested as an explanation without specifying their strength and distribution in the Mid-Atlantic Ridge area. If this were the cue, however, there are magnetic anomalies in the Sargasso Sea too (see^16^) that could be equally useful as signposts.

## Drift simulation with departures from the Mid Atlantic Ridge and from the Sargasso Sea

Chang et al.^1^ modelled the transport of virtual leptocephalus larvae from the intersection of the Mid-Atlantic Ridge by the 22 °C and 24 °C thermal fronts at 27° N and 20° N, respectively, as well as from the Sargasso Sea and concluded that the resulting distribution patterns were very similar. This is not surprising since the modelling of larval drift seems essentially just to reflect the slow westward drift prevailing both in the Sargasso Sea and Mid-Atlantic Ridge areas. The seeding of v-larvae could have been made both east and west of the Mid-Atlantic Ridge area with approximately the same result. Also the assumption of using the intersection of the Mid-Atlantic Ridge by the 22 °C and 24 °C thermal fronts as presumed spawning places seems to have little basis in observations. Usually the 22 °C to 24 °C interval is used to define the SST range where the subtropical convergence frontal zone can be found. As seen in Figure 1 of Chang et al.^1^ this interval diverges towards the east, which means that the convergence zone is weakening. There is no indication in the World Oceanic Atlas data (https://www.nodc.noaa.gov/OC5/woa18/) neither of one nor two temperature fronts at depths where leptocephali are found along a 45 W latitudinal section in the middle of the Mid-Atlantic Ridge area.

## Elevated manganese (Mn) concentrations in the otolith cores of glass eels as a hint for successful spawning only in areas with volcanic activity

Chang et al.^1^ claim that elevated Mn concentrations in the otolith cores of glass eels are a direct proof for successful spawning events in areas of increased volcanic activity, based on observations of Martin et al.^17^, who reported differences of otolith Mn concentrations between *A. anguilla* leptocephalus larvae caught in the Sargasso Sea and the cores of otoliths from glass eels that have arrived in continental waters. However, the results of Martin et al.^17^ were apparently simply misread by Chang et al.^1^. The study of Martin et al.^17^ found higher Mn/Sr ratios in leptocephalus otoliths [1.66E−05 (mean)] from the Sargasso Sea compared to glass eel otoliths from 11 coastal water sites [5.25E−06 to 9.22E−06 (range of means)]. Chang et al.^1^ misquote this study as evidence of a higher Mn concentration in glass eel otoliths compared to leptocephalus larvae from the Sargasso Sea. The hypothesis of higher Mn concentration as an indicator for volcanic activity in the spawning areas is thus just based on an erroneous interpretation of the findings of Martin et al.^17^.

It needs to be added that the underlying hypothesis, that water emerging from hot vents causes an increased Mn concentration locally at the surface, is very unlikely. After an initial rise the plume will mix into the deep-water flow at depths between 2000 and 1000 m, and be advected horizontally. To reach the surface it has to diffuse vertically more than 1000 m through the permanent thermocline. With a typical vertical turbulent eddy diffusion coefficient of the order of magnitude 1–10 cm^2^/s this will take years. Even assuming a very slow horizontal advection the imprint at the ocean surface of the sources will bear no relation to the geography of the ridge. The Mn distribution will essentially be the result of horizontal mixing along isopycnal surfaces. If the concentration of Mn in larval otoliths is a local phenomenon it has to have a source in the surface layer.

Although the incorporation of Mn into otoliths is still not fully understood, Chang et al.^1^ do not discuss and consider other factors that might influence Mn incorporation into otoliths. This disregards the fact that elevated Mn concentrations are often found in otolith primordia (e.g.^18,19^), regardless of the Mn concentrations in the surrounding water^18^. It must therefore be questioned if Mn is suitable for distinguishing among natal sites^20^. Possible reasons for elevated core concentrations in otoliths can be e.g. maternal investment^19^, embryonic development^18^ or variations of crystal structures^18^. Limburg et al.^21,22^ also found elevated Mn concentrations in otoliths of different species caused by hypoxic conditions and water temperature also affects Mn incorporation into otoliths (e.g.^23,24^).

## Insufficient sampling efforts and a limited area coverage of recent surveys as a possible reason for “false negative” observations along the Mid-Atlantic Ridge

Chang et al.^1^ state that the proposed area at the Mid-Atlantic Ridge was probably never properly sampled. This statement does not recognize the investigations by Johannes Schmidt as well as earlier and later surveys in the Mid-Atlantic Ridge area. The ICES “Eggs and Larvae database” records a total of 48 *A anguilla* leptocephali caught within the area 15–29° N and 43–48° W, at 10 stations between 1913 and 1970. This area covers the proposed Mid-Atlantic Ridge area. All leptocephali were large (mean length 41 mm, range 23–45 mm), indicating that they were from last year’s cohort. The ICES database does not include null-stations, which makes it difficult to assess the total survey effort. Schmidt surveyed the area with Dana I in 1920–1921, with 8 null-stations and no leptocephalus caught^16^.

Due to the sheer dimension of the presumed spawning area of anguillid eels in the subtropical convergence zone of the Sargasso Sea, the search for specific spawning sites in space and time by classical fisheries or larval surveys is laborious and costly, resembling the proverbial search of a needle in a haystack. Any approach to narrow the assigned search area down by learning from scientific knowledge on congeneric species like the Japanese eel or modelling exercises can be extremely useful, as it is to more generally challenge allegedly established scientific hypotheses. However, such challenges require great care concerning the underlying data quality and novelty to avoid duplications and the dispersion of scarce resources.

## References

[1] Chang YK, Feunteun E, Miyazawa Y, Tsukamoto K (2020). New clues on the Atlantic eels spawning behavior and area: the mid-Atlantic Ridge hypothesis. Sci. Rep..

[2] Schmidt J (1923). Breeding places and migrations of the Eel. Nature.

[3] Boëtius J, Harding EF (1985). A re-examination of Johannes Schmidt’s Atlantic eel investigations. Dana.

[4] Boëtius J, Harding EF (1985). A list of Atlantic and Mediterranean *Anguilla* leptocephali: Danish material up to 1966. Dana.

[5] Schoth M, Tesch F-W (1982). Spatial distribution of 0-Group eel larvae (*Anguilla* spec.) in the Sargasso Sea. Helgoänder Meeresunters..

[6] Hanel R (2014). Low larval abundance in the Sargasso Sea: New evidence about reduced recruitment of the Atlantic eels. Naturwiss.

[7] Westerberg H (2017). Modelling the drift of European (*Anguilla anguilla*) and American (*Anguilla rostrata*) eel larvae during the year of spawning. Can. J. Fish. Aquat. Sci..

[8] Miller MJ (2019). Spawning by the European eel across 2000 km of the Sargasso Sea. Biol. Lett..

[9] Miller MJ (2015). A century of research on the larval distribution of Atlantic eels: A re-examination of the data. Biol. Rev..

[10] Tesch F-W (2003). The Eel.

[11] Farmer, M. W. Cruise Report W-54: Scientific activities undertaken aboard the R/V Westward, Woods Hole—St. Lucia—Tobago Cays—Bequia—St. Homas, 8 October 1980–19 November 1980, Technical Report. 10.1575/1912/4568. https://hdl.handle.net/1912/4568 (1980).

[12] Fricke H, Tsukamoto K (1998). Seamounts and the mystery of eel spawning. Naturwiss.

[13] Fricke H, Käse R (1995). Tracking of artificially matured eels (*Anguilla anguilla*) in the Sargasso Sea and the problem of the eel’s spawning site. Naturwiss.

[14] Righton D (2016). Empirical observations of the spawning migration of European eels: The long and dangerous road to the Sargasso Sea. Sci. Adv..

[15] Wysujack K (2015). The migration behaviour of European silver eels (*Anguilla anguilla*) released in open ocean conditions. Mar. Freshw. Res..

[16] Perivier, H. A. A spatial and geophysical exploration of Atlantic Eel larval distributions. Master's thesis, Harvard Extension School. https://dash.harvard.edu/handle/1/24078374 (2015).

[17] Martin J (2010). An otolith microchemistry study of possible relationships between the origins of leptocephali of European eels in the Sargasso Sea and the continental destinations and relative migration success of glass eels. Ecol. Freshw. Fish.

[18] Brophy D, Jeffries TE, Danilowicz BS (2004). Elevated manganese concentrations at the cores of clupeid otoliths: Possible environmental, physiological, or structural origins. Mar. Biol..

[19] Ruttenberg BI (2005). Elevated levels of trace elements in cores of otoliths and their potential for use as natural tags. Mar. Ecol. Prog. Ser..

[20] Ben-Tzvi O, Abelson A, Gaines SD (2007). The inclusion of sub-detection limit LA-ICPMS data, in the analysis of otolith microchemistry, by use of a palindrome sequence analysis (PaSA). Limnol. Oceanogr. Methods.

[21] Limburg KE (2015). In search of the dead zone: Use of otoliths for tracking fish exposure to hypoxia. J. Mar. Syst..

[22] Limburg KE, Casini M (2019). Otolith chemistry indicates recent worsened Baltic cod condition is linked to hypoxia exposure. Biol. Lett..

[23] Miller JA (2009). The effects of temperature and water concentration on the otolith incorporation of barium and manganese in juvenile black rockfish (*Sebastes melanops* Girard). J. Fish Biol..

[24] Marohn L (2011). Temperature dependency of element incorporation into European eel (*Anguilla anguilla*) otoliths. Anal. Bioanal. Chem..

